# The NF-kappa B inhibitor, celastrol, could enhance the anti-cancer effect of gambogic acid on oral squamous cell carcinoma

**DOI:** 10.1186/1471-2407-9-343

**Published:** 2009-09-25

**Authors:** Di He, Qin Xu, Ming Yan, Ping Zhang, Xiaojian Zhou, Zhiyuan Zhang, Wenhu Duan, Laiping Zhong, Dongxia Ye, Wantao Chen

**Affiliations:** 1 Department of Oral and Maxillofacial Surgery, College of Stomatology, Ninth People's Hospital, Shanghai Jiao Tong University School of Medicine, Shanghai 200011, PR China; 2 Laboratory of Oral Tumor and Oral Biology, Shanghai Key Laboratory of Stomatology and Shanghai Research Institute of Stomatology, Shanghai 200011, PR China; 3 Department of Medicinal Chemistry, Shanghai Institute of Materia Medica, Chinese Academy of Sciences, Shanghai 201203, PR China; 4 Department of Oral and Maxillofacial Surgery, Second Affiliated Hospital, Zhejiang University School of Medicine, Hangzhou 310009, PR China

## Abstract

**Background:**

Gambogic acid (GA) is a major active ingredient of gamboge, a widely used traditional Chinese medicine that has been reported to be a potent cytotoxic agent against some malignant tumors. Many studies have shown that the NF-kappa B signaling pathway plays an important role in anti-apoptosis and the drug resistance of tumor cells during chemotherapy. In this study, the effects and mechanisms of GA and the NF-kappa B inhibitor celastrol on oral cancer cells were investigated.

**Methods:**

Three human oral squamous cell carcinoma cell lines, Tca8113, TSCC and NT, were treated with GA alone, celastrol alone or GA plus celastrol. Cytotoxicity was assessed by MTT assay. The rate of apoptosis was examined with annexin V/PI staining as well as transmission electronic microscopy in Tca8113 cells. The level of constitutive NF-kappa B activity in oral squamous cell carcinoma cell lines was determined by immunofluorescence assays and nuclear extracts and electrophoretic mobility shift assays (EMSAs) *in vitro*. To further investigate the role of NF-kappa B activity in GA and celastrol treatment in oral squamous cell carcinoma, we used the dominant negative mutant SR-IκBα to inhibit NF-kappa B activity and to observe its influence on the effect of GA.

**Results:**

The results showed that GA could inhibit the proliferation and induce the apoptosis of the oral squamous cell carcinoma cell lines and that the NF-kappa B pathway was simultaneously activated by GA treatment. The minimal cytotoxic dose of celastrol was able to effectively suppress the GA-induced NF-kappa B pathway activation. Following the combined treatment with GA and the minimal cytotoxic dose of celastrol or the dominant negative mutant SR-IκBα, proliferation was significantly inhibited, and the apoptotic rate of Tca8113 cells was significantly increased.

**Conclusion:**

The combination of GA and celastrol has a synergistic antitumor effect. The effect can be primarily attributed to apoptosis induced by a decrease in NF-kappa B pathway activation. The NF-kappa B signaling pathway plays an important role in this process. Therefore, combining GA and celastrol may be a promising modality for treating oral squamous cell carcinoma.

## Background

Despite improvements in surgery and radiation, the overall survival rate of patients with oral squamous cell carcinoma (OSCC) has not improved over the past two decades[1]. Chemotherapy (pre- or post-surgery) does not appear to be beneficial for local control and survival improvement in the patients with OSCC. Current chemotherapeutic agents have limited efficacy in oral cancer. To overcome this problem, multiple chemotherapeutic agents with different modes of action, used either alone or in combination, have been suggested[2].

Gambogic acid (GA) is a major active ingredient of gamboge, which has been widely used in traditional Chinese medicine. It is reported that GA possesses diverse biological effects, such as anti-oxidant and anti-infectious activities[3]. Recent pharmacological studies have revealed that GA also has potent cytotoxic and anti-cancer activities in several cancer cell lines [4-8]. However, little is known about the effect of GA on OSCC. In the present study, we investigated and proved that the NF-kappa B pathway was highly activated by GA treatment while inducing cell apoptosis in OSCC. The minimal cytotoxic dose of celastrol effectively suppressed the GA-induced NF-kappa B pathway activation and increased the anti-cancer effect of GA.

## Methods

### Reagents

GA and celastrol were provided by Calbiochem (Germany). Dimethyl sulfoxide (DMSO) was purchased from Sigma Chemical Company (USA). The compounds were dissolved in DMSO to generate a stock concentration of 100 mM. Subsequent dilutions were made in culture medium. The same proportion of DMSO/culture medium was added to the controls. The final DMSO content was less than 0.1%. RPMI 1640 culture medium and fetal bovine serum (FBS) were obtained from Gibco (USA). 3'-(4,5-dimethylthiazol-2-yl)-2, 5-diphenyl tetrazolium bromide (MTT), bovine serum albumin (BSA) and Hoechst 33342 were purchased from Sigma Chemical Company. PI/RNase Staining Buffer and the Annexin V-FITC Apoptosis Detection Kit I were purchased from BD Pharmingen (USA). The mouse monoclonal antibody against NF-kappa B p65 was purchased from Santa Cruz Biotechnology Incorporation (USA). NE-PER nuclear and cytoplasmic extraction reagents and the LightShift-TM chemiluminescent EMSA kit were purchased from Pierce Biotechnology (USA). Lipofectamine 2000 reagent was purchased from Invitrogen (USA).

### Cell lines

Three human oral squamous cell carcinoma cell lines were included in the study. Tca8113 was established in our laboratory. TSCC was a gift from the School of Stomatology, Wuhan University, China. NT was a gift from Nagasaki University, Japan. Cells were cultured in RPMI 1640 medium supplemented with 10% (v/v) FBS, 100 units/ml penicillin and 100 units/ml streptomycin at 37°C in a humidified atmosphere containing 5% CO_2 _[9,10].

### Plasmid and transient transfection

Cells were plated in 6-well plates and transfected with pBabe-SR-IκBα or pBabe using Lipofectamine 2000 reagents according to the manufacturer's instructions (Invitrogen Life Technologies). Six hours after transfection, the complex medium was replaced with RPMI 1640 medium supplemented with 10% (v/v) FBS. After an additional 24 hours of incubation, cells were treated with or without GA.

### Cell viability assay

Cells were plated into a 96-well plate at a density of 1 × 10^3^/well and treated with the indicated dose of GA, celastrol or both after 24 hours of incubation. For the cell viability assay, 20 μl of MTT dissolved in PBS (5 mg/ml) was added to each well. The plates were further incubated for 4 hours. The absorbance of each well was measured using an enzyme-linked immunosorbent assay reader at 490 nm after being dissolved in 150 μl of DMSO.

### Transmission electron microscopy

Cells were cultured with or without 4 μM GA for 48 hours and then immersed in cacodylate-buffered 2.5% glutaraldehyde at 4°C for 3 hours. Subsequently, cells were washed for 1 hour in 0.1 M cacodylate buffer, pH 7.4, fixed for 1 hour in buffered 1% osmium tetroxide, dehydrated in graded ethanol and embedded in Epon 812. Thin sections (600 Å thick) were stained with uranyl acetate and lead citrate and then observed with a Zeiss EM 900 transmission electron microscopy (Zeiss, Germany) at 50 kV.

### DNA content and cell cycle analysis

Cells were treated with 4 μM GA for different periods, trypsinized, washed once with PBS and fixed in 70% ethanol for 2 hours at 4°C. After washing with PBS, fixed cells were incubated with 0.5 ml PI/RNase staining buffer for 15 minutes at room temperature. DNA content and cell cycle status were assessed by FACScan laser flow cytometry (FACSCalibur, Becton Dickinson, USA). The data were analyzed using MODFIT and CELLQUEST software. Apoptotic cells were distinguished from non-apoptotic intact cells by their decreased DNA content, as determined by their lower PI staining intensity in the area below the sub-G0/G1 phase.

### Annexin V/PI double-staining assay

Cells (1 × 10^6^) were treated with the indicated dose of GA, celastrol or a combination of both. Apoptotic cells were identified by double supravital staining with recombinant FITC-conjugated annexin V and PI using the Annexin V-FITC apoptosis detection kit according to the manufacturer's instructions. Flow cytometry analysis was performed immediately after supravital staining. Data acquisition and analysis were performed by a Becton Dickinson FACSCalibur flow cytometer using CellQuest software. Cells that were annexin V (-) and PI (-) were considered viable cells. Cells that were annexin V (+) and PI (-) were considered early-stage apoptotic cells. Cells that were annexin V (+) and PI (+) were considered late-stage apoptotic cells. Cells that were annexin V (-) and PI (+) were considered necrotic cells.

### Immunofluorescence

Cells were grown on coverslips and cultured for 24 hours. After treatment with the indicated doses of GA, celastrol or both for 12 hours, cells were fixed in 4% paraformaldehyde for 15 minutes, washed in PBS and treated for 15 minutes with PBS containing 0.1% Triton X-100. The cells were then washed, blocked with 10% bovine serum albumin (BSA) for 1 hour at room temperature and incubated at 4°C overnight with NF-kappa B p65 antibody (1:100) diluted in 0.1% BSA. After extensive washing, a 1:200 dilution of FITC-conjugated goat anti-mouse IgG was applied as the secondary antibody for 1 hour at room temperature. Nuclear staining was achieved by incubating cells in Hoechst 33342 for 5 minutes. The slides were then washed and photographed with a laser scanning confocal microscope (TCS SP2, Leica, Germany). The positive control was treated with 50 ng/ml TNF-α (Sigma-Aldrich, USA) for 30 minutes.

### Nuclear extracts and electrophoretic mobility shift assays (EMSAs)

Cells (1 × 10^6^) were treated with the indicated doses of GA, celastrol or both, and the nuclear extracts were prepared using NE-PER nuclear and cytoplasm extraction reagents according to the manufacturer's instructions. Synthetic complementary oligonucleotides were 3'-biotinylated using the biotin 3'-end DNA labeling kit (Pierce) according to the manufacturer's instructions and annealed for 2 hours at room temperature. The sequences of the oligonucleotides for NF-kappa B were: 5' - AGT TGA GGG GAC TTT CCC AGG C - 3' and 3' - TCA ACT CCC CTG AAA GGG TCC G - 5'. The binding reaction was carried out for 20 minutes at room temperature in the presence of 50 ng/μl poly (dI-dC), 0.05% Nonidet P-40, 5 mM MgCl2, 10 mM EDTA and 2.5% glycerol in 1× binding buffer (LightShift-TM chemiluminescent EMSA kit), using 20 fM biotin-end-labeled target DNA and 5 μg of nuclear extract. Products were loaded onto native 4% polyacrylamide gels, pre-electrophoresed for 60 minutes in 0.5× Tris borate/EDTA and then submitted to electrophoresis at 100 V before being transferred onto a positively charged nylon membrane (HybondTM-N+) in 0.5× Tris borate/EDTA at 100 V for 30 minutes. Transferred DNA was cross-linked to the membrane at 120 mJ/cm^2 ^and detected using horseradish peroxidase-conjugated streptavidin (LightShift-TM chemiluminescent EMSA kit), according to the manufacturer's instructions.

### Statistical analysis

All assays were repeated three times to insure reproducibility. The significance of results obtained from the control and treated groups was analyzed using the paired Student's t-test. The synergistic effects of GA and celastrol were assessed using factorial ANOVA. Means and standard deviations were calculated. *P *< 0.05 was regarded as statistically significant.

## Results

### Effects of GA on cell viability

**Figure 1:**
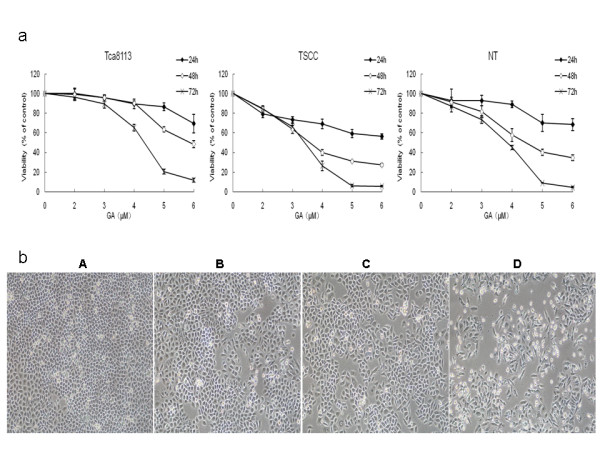
**Effects of GA on the viability of oral squamous cell carcinoma cell lines**. a: Three oral squamous carcinoma cell lines were treated with 0, 2, 3, 4, 5 and 6 μM of GA for 24 hours, 48 hours and 72 hours. The inhibitory effects of GA were shown in a time- and dose-dependent manner. b: Morphological changes of Tca8113 cells under phase contrast microscopic observation induced by 4 μM GA from 0 to 72 hours. (A: 0, B: 24 hours, C: 48 hours, D: 72 hours, 100×, original magnification).

The MTT assay was performed to analyze the effects of GA on the viability of Tca8113, TSCC and NT cells. As shown in Figure 1, GA inhibited the growth of these three cell lines in both a time- and dose-dependent manner. Over time, more and more cells began to shrink, indicating increased cell death induced by GA treatment. The IC50 values (the concentration of GA that results in a 50% reduction in absorbance compared with the control) for these three cell lines after 72 hours of treatment were calculated based on the MTT assay. The IC50 of GA was 4.23 μM for Tca8113, 3.34 μM for TSCC and 3.76 μM for NT cells.

### Apoptotic changes following GA treatment

**Figure 2:**
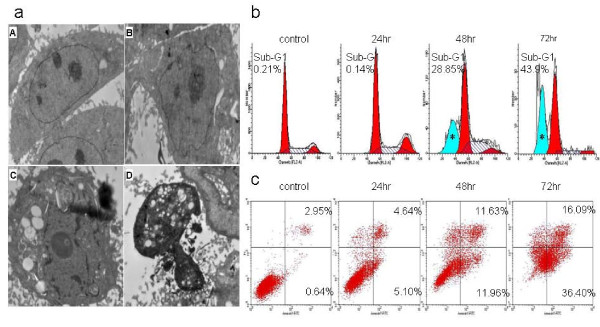
**Apoptotic changes in GA-treated OSCC cells**. a: Morphological changes of Tca8113 cells under transmission electronic microscopy (A: control, B: early-stage apoptosis, C: middle-stage apoptosis, D: late-stage apoptosis, 4000 × 10 original magnification). b: Flow cytometry analysis of DNA content in Tca8113 cells treated with 4 μM GA for 24 hours to 72 hours (*: the sub-G1 peak). Percentages of sub-G1 peak are labeled. c: The rates of cell apoptosis were measured by annexin V and PI staining and detected by flow cytometry. Percentages of early apoptosis (Lower Right region) and late apoptosis (Upper Right region) are labeled. Images are representative of three independent experiments.

To elucidate whether the growth inhibitory effect of GA was attributable to the induction of apoptosis, transmission electron microscopy, cell cycle analysis and flow cytometric analysis with annexin V/PI double staining were performed. First, transmission electron microscopy was used to determine the subcellular changes evoked by GA in Tca8113 cells. We observed that the nuclei of cells exposed to 4 μM GA for 48 hours were pyknotic and that some nuclei were fragmented. There were various vacuoles and "apoptotic bodies" in the intra-cytoplasm or extra-cytoplasm (Figure 2a). Secondly, the cell cycle analysis of Tca8113 cells treated with 4 μM GA for 48 hours and 72 hours demonstrated a distinct population of cells with DNA content below the G1 phase (a sub-G1 peak), indicating the presence of apoptotic cells (Figure 2b). Finally, flow cytometric analysis with annexin V/PI double staining revealed that treatment with 4 μM GA significantly increased the percentage of both early and late apoptotic cells in a time-dependent manner (Figure 2c) (*P *< 0.05).

### Activation of the NF-kappa B pathway in GA-induced apoptosis

**Figure 3:**
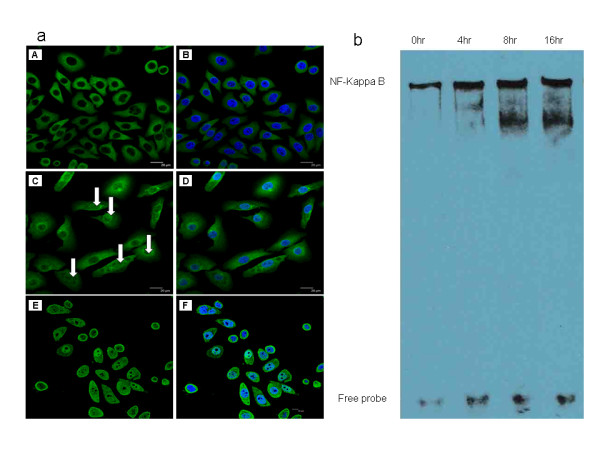
**Activation of NF-kappa B pathway in GA-induced apoptosis**. a: Nuclear translocation of p65 subunit of NF-kappa B in Tca8113 cells after treatment with GA or TNF-α (A: control, without nuclear staining, B: control, with nuclear staining, C: 12 hours treatment with 4 μM GA, without nuclear staining, D: 12 hours treatment with 4 μM GA, with nuclear staining, E: 60 minutes treatment with 50 ng/ml TNF-α, without nuclear staining, F: 60 minutes treatment with 50 ng/ml TNF-α, with nuclear staining). b: EMSA analysis of NF-kappa B binding activity in Tca8113 cells after 4 to 16 hours of treatment with 4 μM GA.

To determine the effect of GA on the activity of the NF-kappa B pathway in OSCC cells, the nuclear translocation of the cytoplasmic NF-kappa B p65 subunit was examined in Tca8113 cells by immunostaining. The results showed that 12 hours of GA treatment stimulated NF-kappa B p65 nuclear translocation, as did 50 ng/ml TNF-α treatment for 1 hour (Figure 3a). After GA treatment for 4, 8 and 16 hours, the effect of GA on the binding activity of NF-kappa B to its consensus DNA oligonucleotide was also examined by EMSA. As indicated in Figure 3b, GA treatment caused a significant increase in NF-kappa B DNA binding activity.

### Synergistic effects of celastrol on GA-induced cytotoxicity

**Figure 4:**
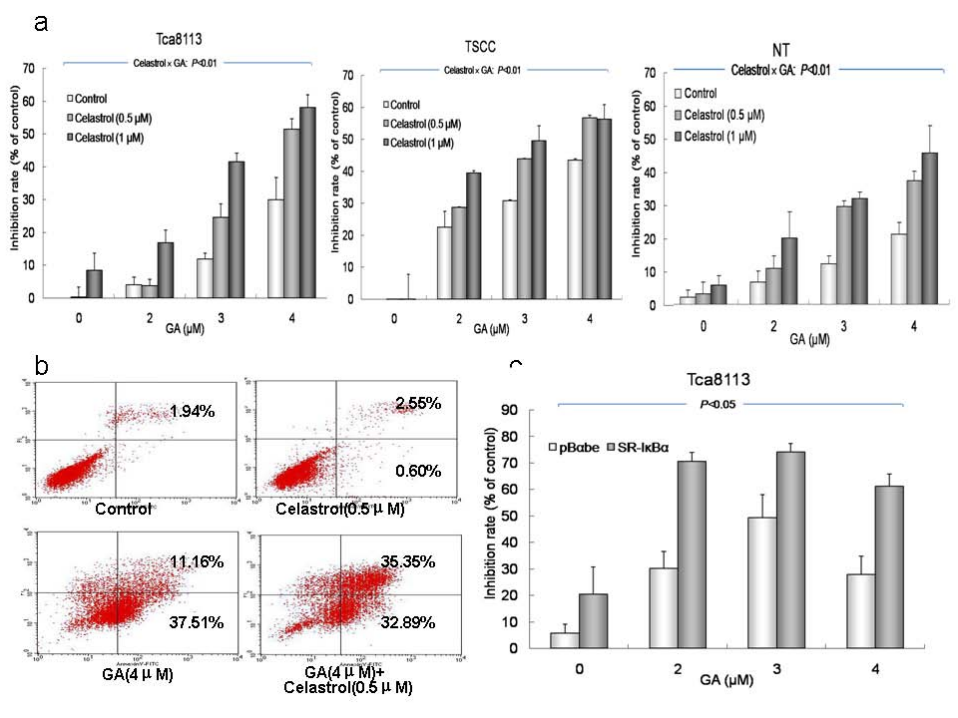
**Synergistic effect of celastrol on GA-induced cytotoxicity**. a: Inhibitory effects of combined treatment with GA and celastrol on three oral squamous cell carcinoma cell lines. Cells were treated with different doses of GA (0, 2, 3 and 4 μM) and celastrol (0, 0.5 and 1 μM) for 72 hours. The interaction effects of GA and celastrol on all three cell lines were highly significant (*P *< 0.01, factorial ANOVA). b: Annexin V and PI staining of Tca8113 cells after treatment with 4 μM GA and 0.5 μM celastrol for 72 hours. The increase in apoptotic cells in combined treatment group was significant (*P *< 0.05, Student's t-test), compared with cells treated with GA alone. c: Inhibitory effects of Tca8113 cells after transient transfection with the dominant mutant pBabe-SR-IκBα (specific NF-Kappa B inhibitor) for 24 hours and treatment with 4 μM GA for 72 hours. Images are representative of three independent experiments.

GA activated the NF-kappa B signaling pathway while inducing cell apoptosis. A minimal dose of the NF-kappa B inhibitor celastrol was used to block the GA-induced activation of NF-Kappa B, with the goal of enhancing the anti-cancer effect of GA on OSCC cells. As Figure 4a shows, celastrol can effectively enhance the cytotoxicity of GA in all three cell lines. The combined effects of celastrol and GA on all of the cell lines were significant (*P *< 0.01), as evaluated by factorial ANOVA.

Annexin V/PI double staining indicated that the enhanced cytotoxicity of GA and celastrol resulted from increased apoptosis in Tca8113 cells. Treatment with 0.5 μM celastrol alone did not induce a remarkable degree of cell apoptosis, but upon co-treatment with GA, the number of apoptotic cells significantly increased (*P *< 0.05 compared to cells treated with GA alone; Figure 4b). Similarly, when GA was combined with a specific NF-kappa B inhibitor, the dominant negative mutant pBabe-SR-IκBα, the proliferation of Tca8113 cells was significantly inhibited compared to cells treated with GA combined with pBabe empty vector (Figure 4c).

### Effects of celastrol on GA-induced activation of NF-kappa B

**Figure 5:**
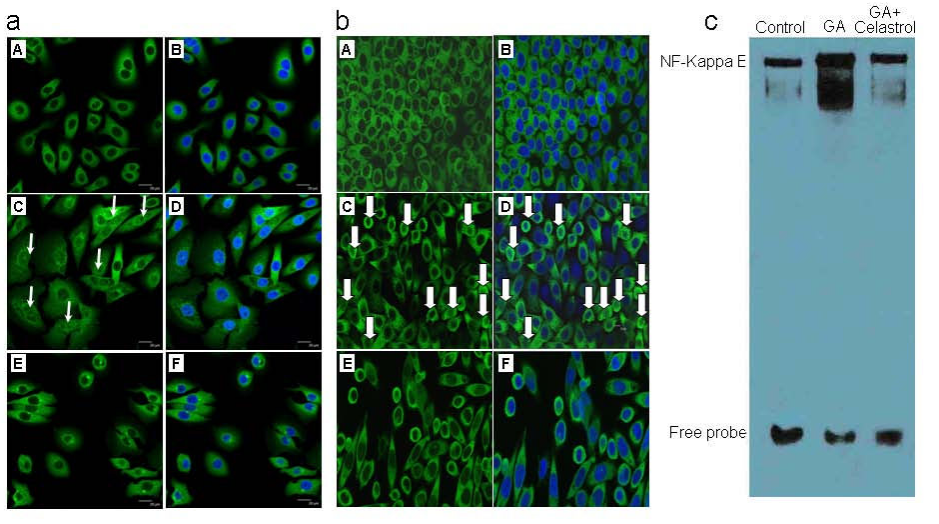
**Effects of celastrol on GA-induced activation of NF-kappa B**. a: Nuclear translocation of the p65 subunit of NF-kappa B in Tca8113 cells after 12 hours of treatment with 4 μM GA and 0.5 μM celastrol (A: control, without nuclear staining, B: control, with nuclear staining, C: 12 hours after treatment with 4 μM GA alone, without nuclear staining, D: 12 hours after treatment with 4 μM GA alone, with nuclear staining, E: 12 hours after combined treatment with 4 μM GA and 0.5 μM celastrol, without nuclear staining, F: 12 hours after combined treatment with 4 μM GA and 0.5 μM celastrol, with nuclear staining). b: Nuclear translocation of the p65 subunit of NF-kappa B in Tca8113 cells after a 24 hours transient transfection of the dominant mutant pBabe-SR-IκBα and 12 hours treatment with 4 μM GA. (A: control, without nuclear staining, B: control, with nuclear staining, C: 24 hours transfection with pBαbe and 12 hours treatment with 4 μM GA, without nuclear staining, D: 24 hours transfection with pBabe and 12 hours treatment with 4 μM GA, with nuclear staining, E: 24 hours transfection with pBabe-SR-IκBα and 12 hours treatment with 4 μM GA, without nuclear staining, F: 24 hours transfection with pBabe-SR-IκBα and 12 hours treatment with 4 μM GA, with nuclear staining). c: EMSA analysis of NF-kappa B binding activity in Tca8113 cells after 12 hours of treatment with 4 μM GA and 0.5 μM celastrol (A: control, B: after treatment with GA alone, C: after combined treatment with GA and celastrol).

To determine whether the synergistic effect of celastrol was mediated by suppression of the GA-induced NF-kappa B activation, nuclear translocation of the NF-kappa B p65 subunit was examined by immunofluorescence after treatment with GA, GA plus celastrol and GA plus pBabe-SR-IκBα. As Figure 5a and 5b show, both celastrol and pBabe-SR-IκBα effectively blocked the GA-induced nuclear translocation of the p65 subunit. The inhibition of GA-induced NF-kappa B activity by celastrol was further confirmed by EMSA (Figure 5c).

## Discussion

Gambogic acid is a major ingredient of gamboge, a brownish-orange resin exuded from the Asian *Garcinia hanburryi *tree. The resin has been used as Chinese traditional medicine in China for hundreds of years to treat cancers with little side effect. Recent experiments have demonstrated that GA exhibits a general inhibitory effect against various tumor cell lines [4-6]. However, little information is available in the literature about the effect and mechanism of this compound on the growth of human oral squamous cell carcinomas. In this study, we investigated the effects of GA on the growth of human OSCC cell lines. Our results demonstrate that GA significantly inhibits the viability of OSCC cells and induces their apoptosis in a time- and dose-dependent manner. These results suggest that GA could be a potential chemotherapeutic agent, like many other plant-derived natural compounds [11].

The transcription factor, NF-kappa B, is well established as a regulator of genes encoding cytokines, cytokine receptors and cell adhesion molecules that drive immune and inflammatory responses[12]. In recent years, NF-kappa B activation has been linked to many aspects of tumorigenesis[13,14], including the control of apoptosis, cell cycle, differentiation, cell adhesion, cell migration and angiogenesis. Five NF-kappa B family members have been identified in mammalian cells, including p105/p50 (NF-kappa B1), p100/p52 (NF-kappa B2), p65 (REL A), c-REL and RELB, which associate with one another to form various heterodimeric and homodimeric combinations. These proteins are regulated by inhibitors that bind to the inhibitory subunit of the NF-kappa B (I-kappa B) family of ankyrin domain-containing proteins, which include I-kappa B α, I-kappa B β, I-kappa B γ and I-kappa B ε[12]. In a classic NF-kappa B signaling pathway, NF-kappa B resides in the cytoplasm in an inactive state as a heterotrimer consisting of p50, p65 and I-kappa B α subunits. Most carcinogens, inflammatory agents and tumor promoters have been shown to activate NF-kappa B. In response to an activation signal, the I-kappa B α subunit is phosphorylated at serine residues 32 and 36, ubiquitinated at lysine residues 21 and 22, and degraded through the proteasome pathway, thus exposing the nuclear localization signals on the p50-p65 heterodimer. The p65 subunit is then phosphorylated, leading to nuclear translocation and binding to a specific sequence in DNA, which, in turn, results in gene transcription[15]. NF-kappa B regulates the expression of several genes whose products are involved in anti-apoptosis such as Bcl-xL, cIAP and TRAF[16,17].

It has been found that many chemotherapeutic agents, including taxol, doxorubicin, etoposide, cisplatin and tamoxifen, can activate NF-kappa B[18]. The activation of NF-kappa B can lead to resistance to apoptosis induced by chemotherapeutic agents. Thus, while activating apoptosis, the same agent can also activate NF-kappa B[19,20], which can prevent apoptosis and block the ability of therapeutic agents to induce cell death. The recognized method of GA's antitumor effect is its ability to bind and interact with the transferrin receptor, and thus induce cell apoptosis[8]. It is also suggested that GA has the ability to repress telomerase activity, which inhibits the proliferation of tumor cells[5]. In this study, we examined the effect of GA on the activation of NF-kappa B in OSCC cells. The results clearly show that GA stimulates nuclear translocation of NF-kappa B p65, up-regulates the DNA binding activity of NF-kappa B, and thus activates the NF-kappa B signaling pathway. Considering the role of NF-kappa B in preventing apoptosis, we could hypothesize that, when treated with GA, an apoptosis inducer, OSCC cells activate the NF-kappa B signaling pathway to resist the apoptosis-inducing effect. This process is similar to the chemotherapy resistance caused by other chemotherapeutic agents.

The pivotal role of the NF-kappa B pathway in the inhibition of cell apoptosis, strongly suggests that NF-kappa B inhibitors would be useful in cancer therapy. In recent years, it has been reported that some agents, mostly plant-derived products, with tumor chemopreventative and chemotherapeutic effects suppress the activation of NF-kappa B[21]. Although some of these agents are known to induce apoptosis, the most likely therapeutic consequence of NF-kappa B inhibition is a reduced apoptotic threshold. Therefore, a reasonable strategy is to use a NF-kappa B inhibitor as an adjuvant with other modalities to sensitize tumor cells to chemotherapeutic agents.

Celastrol is a novel compound that has demonstrated the ability to inhibit cancer progression and down-regulate NF-kappa B activity. It is also a natural product extracted from the traditional Chinese medicine "Thunder of God Vine"[22]. Celastrol has been shown to decrease NF-kappa B activity in prostate cancer and leukemia cells[23,24]. However, it had not previously been evaluated as a potential chemotherapeutic sensitizer for head and neck or other solid tumors. Our results show that a low dose of celastrol (less than 2 μM) had no significant cytotoxic effect on OSCC cells. However, celastrol had a significant synergistic effect on GA-induced apoptosis in OSCC cells. Our combined results show that celastrol decreases NF-kappa B activity stimulated by GA and then decreases the apoptotic threshold of OSCC cells and sensitizes cells to GA-induced apoptosis by inhibiting the NF-kappa B signaling pathway.

In most cases, the efficacy of chemotherapeutic agents is limited due to toxicity and side effects. Therefore, increasing the potency of chemotherapeutic agents remains a worthy goal. Our results show that GA demonstrates antitumor and apoptosis-inducing abilities in oral squamous cell carcinoma cells. GA activates the NF-kappa B pathway while inducing apoptosis, thus blunting its own antitumor effect. Celastrol has a synergistic effect on the GA-induced apoptosis in oral cancer cells by inhibiting the NF-kappa B activity stimulated by GA.

## Conclusion

Our study suggests that a minimally toxic dose of celastrol could enhance the cytotoxicity and apoptosis-inducing effect of GA. These results indicate that GA's antitumor effect could be amplified in a minimally toxic way by inhibiting NF-kappa B activity in OSCC cells. Celastrol may also be useful in combination therapies by enhancing the efficacy of chemotherapeutic agents with little toxicity or side effects.

## Competing interests

The authors declare that they have no competing interests.

## Authors' contributions

WC and PZ were responsible for the study design, interpretation of the data and revision of the manuscript. DH and MY were responsible for data acquisition, analysis of the work presented and the preparation of the manuscript. QX, DY and XZ participated in the study of growth inhibition, cell apoptosis and determination of NF-kappa B activity. WD, ZZ and LZ supervised the studies and helped to revise the manuscript. All authors read and approved the final manuscript.

## Pre-publication history

The pre-publication history for this paper can be accessed here:

http://www.biomedcentral.com/1471-2407/9/343/prepub
